# Sharpness of Spike Initiation in Neurons Explained by Compartmentalization

**DOI:** 10.1371/journal.pcbi.1003338

**Published:** 2013-12-05

**Authors:** Romain Brette

**Affiliations:** 1 Laboratoire Psychologie de la Perception, CNRS and Université Paris Descartes, Paris, France; 2 Equipe Audition, Département d'Etudes Cognitives, Ecole Normale Supérieure, Paris, France; The University of Chicago, United States of America

## Abstract

In cortical neurons, spikes are initiated in the axon initial segment. Seen at the soma, they appear surprisingly sharp. A standard explanation is that the current coming from the axon becomes sharp as the spike is actively backpropagated to the soma. However, sharp initiation of spikes is also seen in the input–output properties of neurons, and not only in the somatic shape of spikes; for example, cortical neurons can transmit high frequency signals. An alternative hypothesis is that Na channels cooperate, but it is not currently supported by direct experimental evidence. I propose a simple explanation based on the compartmentalization of spike initiation. When Na channels are placed in the axon, the soma acts as a current sink for the Na current. I show that there is a critical distance to the soma above which an instability occurs, so that Na channels open abruptly rather than gradually as a function of somatic voltage.

## Author Summary

Spike initiation determines how the combined inputs to a neuron are converted to an output. Since the pioneering work of Hodgkin and Huxley, it is known that spikes are generated by the opening of sodium channels with depolarization. According to this standard theory, these channels should open gradually when the membrane potential increases, but spikes measured at the soma appear to suddenly rise from rest. This apparent contradiction has triggered a controversy about the origin of spike “sharpness.” This study shows with biophysical modelling that if sodium channels are placed in the axon rather than in the soma, they open all at once when the somatic membrane potential exceeds a critical value. This work explains the sharpness of spike initiation and provides another demonstration that morphology plays a critical role in neural function.

## Introduction

Action potentials are generated in central neurons by the opening of sodium channels in the axon initial segment (AIS) [1]. From patch-clamp studies, it is known that these channels open gradually with depolarization, with a Boltzmann slope factor of about 6 mV [2], [3]. Yet the onset of spikes recorded at the soma of cortical neurons appears very sharp, much sharper than would be expected in an isopotential membrane, according to standard biophysics [4]. There is a distinct “kink” at spike onset, which appears in a voltage trace as a rapid voltage transition from the resting membrane potential. This kink has been explained by the “lateral current hypothesis”: spikes are initiated in the axon and backpropagated to the soma, so that the kink reflects the sharpened current coming from the axon [5], [6], an observation already made in the early days of electrophysiology [7]. In particular, the phenomenon can be replicated in multicompartmental models based on standard Hodgkin-Huxley formalism [8], [9], provided sodium channel density is high enough in the AIS [10].

However, this explanation misses an important part of the story, because it focuses on the shape of action potentials, rather than on spike initiation per se. Indeed several lines of evidence indicate that spike initiation is very sharp, and not only the initial shape of spikes seen at the soma. First, cortical neurons can reliably transmit frequencies up to 200–300 Hz, and respond to input changes at the millisecond timescale [11], [12]. This is surprising because theoretical studies predict this effect for integrate-and-fire models [13], which have sharp spike initiation, but not for isopotential Hodgkin-Huxley models [14]. It was indeed shown that the cut-off frequency of signal transmission in the latter type of models is inversely related to the activation slope factor of Na channels. On this basis, the cut-off frequency should be one order of magnitude lower than empirically observed. Second, current-voltage relationships measured at the soma *in vitro* show an effective slope factor of about 1 mV, instead of the expected 6 mV [15]. Third, spiking responses of cortical neurons to noisy currents injected at the soma are surprisingly well predicted by integrate-and-fire models [16], and when models with parameterized initiation sharpness are optimized to predict these responses, the optimal slope factor is indistinguishable from 0 mV [17].

These remarks imply that sharpness is a functionally relevant property of spike initiation rather than a measurement artifact. In fact, there are two distinct sets of observations. The first set focuses on the shape of spikes at onset, the “kink” seen at the soma in the temporal waveform of the action potential. I will simply refer to this phenomenon as the “kink” at spike onset, that is, the abrupt voltage transition seen at the soma at spike onset. Observations of the second set do not refer to the shape of spikes, but rather to the input-output properties of the spike initiation process. Sharpness of spike initiation refers to the abrupt opening of Na channels at the initiation site when a threshold somatic voltage is exceeded. Thus, it can be quantified as the somatic voltage interval over which available Na channels switch from mostly closed to mostly open: in a single-compartment Hodgkin-Huxley model, it would be on the order of 6 mV; in an integrate-and-fire model, it would be 0 mV (no Na current flows until a spike is generated); in a cooperative Na channel model, it would be in between. Thus, to claim that spike initiation is sharp essentially means that spikes are initiated as in an integrate-and-fire model: a negligible amount of Na current flows until a threshold somatic voltage value is reached and a spike is suddenly produced.

Sharpness of spike initiation and the “kink” at spike onset are directly related in a single-compartment Hodkgin-Huxley model, but they are not necessarily equivalent in a spatially extended neuron. Using a simple geometrical model consisting of a sphere and a thin cylinder, I give a parsimonious account of these observations by showing that spike initiation sharpness arises from the geometrical discontinuity between the soma and the AIS, rather than from backpropagation of spikes. When Na channels are placed in the thin axon, they open abruptly rather than gradually as a function of somatic voltage, as an all-or-none phenomenon. I further show that the phenomenon is governed by equations (a bifurcation) that are mathematically almost equivalent to the cooperativity model of Na channels [4], [18], even though the neuron model follows the standard Hodgkin-Huxley formalism. I then show the relationship between spike initiation sharpness and the shape of spikes at the initiation site and at the soma.

## Results

### Sharpness of spike initiation

**Figure 1:**
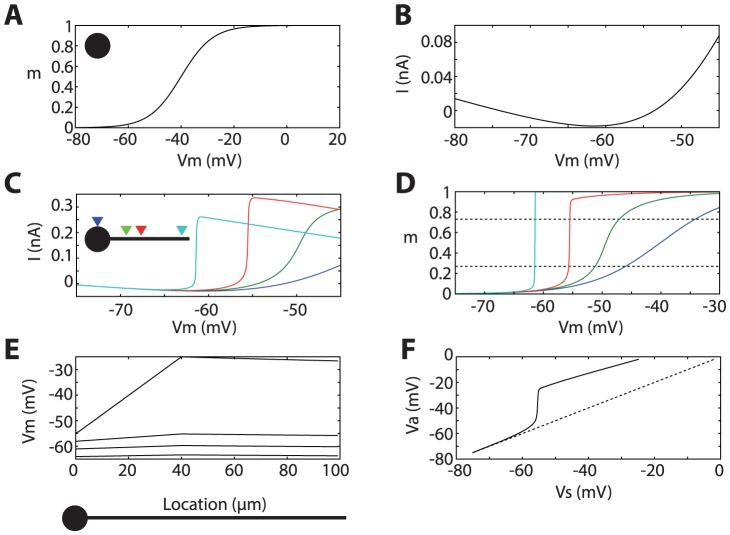
Sharpness of spike initiation with distal initiation (Na channels clustered at a single point). A, Proportion of open Na channels as a function of the voltage of an isopotential neuron. B, Current-voltage relationship in the isopotential neuron with Na and leak channels. C, Current-voltage relationship in a ball-and-stick model, with Na channels at the soma (dark blue), and at 20 µm (green), 40 µm (red) and 100 µm (light blue) away from the soma. D, Proportion of open Na channels as a function of somatic voltage of the ball-and-stick model (color code as in C). E, Voltage across the axon as the soma is depolarized by steps of 3 mV (4 values from −64 mV to −55 mV), with Na channels at 40 µm from the soma. F, Voltage at the initiation site as a function of somatic voltage, showing the loss of voltage control around −55 mV.

In order to clearly demonstrate the phenomenon and avoid confounding factors, I consider a neuron model with only passive leak channels and Na channels (low-threshold Kv1 channels are considered in the Text S1, section 6). The activation curve of Na channels is a Boltzmann function, with half-activation voltage V_1/2_ = −40 mV and slope factor k_a_ = 6 mV (Fig. 1A), consistently with measured properties of Nav1.6 channels in the AIS [9], [19]. Neither Na channel inactivation nor potassium channels were included, so as to isolate the mechanisms responsible for spike initiation and avoid confounding factors (these two factors contribute to repolarization and spike threshold adaptation). In an isopotential neuron, the current-voltage relationship (I–V curve), as measured by a voltage-clamp recording, is well described below V_1/2_ by the sum of a linear part, representing the leak current, and of an exponential part, representing the Na current [14], [20] (Fig. 1B). The minimum of the curve is reached at a voltage value V_T_: this is the maximum voltage that can be reached with a constant current injection without triggering a spike. This voltage is set by V_1/2_ and by the maximal conductance of the Na channel, relative to the leak conductance [21]. The curvature of the exponential function is the slope factor, equal to k_a_, and sets the sharpness of spike initiation, as assessed for example by the cut-off frequency of signal transmission [11], [14].

However, neurons are not isopotential and spikes are initiated in the AIS, not in the soma. In cortical neurons, the AIS is about 1 µm in diameter, and extends from the axon hillock over an unmyelinated length of 10–60 µm [22]. Spikes are initiated about 20–40 µm from the soma [22]. Na channels of the Nav1.6 subtype, which have a low half-activation voltage [19], are concentrated in the AIS, more specifically in the distal part [9]. Thus I now consider a simple geometrical model of the neuron consisting of a sphere (diameter: 50 µm) and a thin cylinder, with a diameter of 1 µm. The cylinder extends over a long distance (300 µm) to avoid artifactual boundary effects. Many neurons also express Na channels in the soma, but since these are not involved in spike initiation, they were not included in the model (except in section “The initial shape of spikes” below; they are responsible for the biphasic nature of phase diagrams [8]). Na channels are placed at a single point in the axon, which we shall call the initiation site. The I–V curve measured at the soma now reflects the sum of the leak current and of the lateral current coming from the axon (Fig. 1C). As is shown in Fig. 1C, an interesting phenomenon occurs as the initiation site is moved away from the soma, while keeping the same maximal Na conductance: the I–V curve becomes sharper, and the voltage at the minimum of the curve also decreases (from −61 mV at the soma to −65 mV at 100 µm away). At 40 µm away from the soma (red), the I–V curve appears indeed much sharper than when Na channels are at the soma (blue).

The increased sharpness due to the lateral current coming from the initiation site has been attributed to the active backpropagation of the distally initiated spike [5], [8]. However, the initiation site is only a small fraction of the axon's space constant away from the soma (which is 700 µm in this model). As a matter of fact, in the present model, there is no active backpropagation since all channels are concentrated at a single location. Fig. 1D shows that the sharpness of the I–V curve directly reflects the abrupt opening of Na channels at the initiation site as the somatic voltage is increased. I now quantitatively define the sharpness of spike initiation as half the somatic voltage interval over which the proportion of open Na channels rises from 27% to 73% (dashed lines). When Na channels are at the soma, this quantity equals k_a_, the Boltzmann slope factor of the Na activation curve (6 mV in this model, in accordance with patch-clamp measurements [9], [19]). But this quantity drops to 2 mV when channels are 20 µm away from the soma, and to 0.1 mV at 40 µm (0.03 mV at 100 µm). In other words, when channels are located at 40 µm away from the soma, they open essentially in an all-or-none fashion when the somatic voltage is increased above a threshold value.

This phenomenon is not due to active backpropagation, since sharpness is defined for Na channels at the initiation site; in addition there are no Na channels between the soma and the initiation site in the present model. Fig. 1E shows what happens along the axon as the soma is depolarized by steps of 3 mV, from −64 mV to −55 mV. At low voltages, the axon is effectively space clamped: the voltage along the axon is essentially equal to the somatic voltage. Indeed cable theory shows that, when Na channels are closed and for an infinite cylindrical axon, voltage decays exponentially along the axon with a space constant of magnitude 1 mm (700 µm in this model), which is essentially constant at this spatial scale. For a shorter cylindrical cable (300 µm in these simulations), voltage decay is even slower. At some critical voltage, Na current flows through the membrane at the initiation site. Because the soma is large compared to the axon, it acts as a current sink: most Na current flows to the soma. This can be seen in the top curve in Fig. 1E, where lateral currents are proportional to the slope: there is a large slope towards the soma, and a horizontal slope towards the distal end of the axon. As a result, there is a loss of space clamp: the voltage now peaks at −25 mV at the initiation site, while it is −55 mV at the soma. Fig. 1F shows the voltage at the initiation site as a function of the somatic voltage. At about −56 mV, a loss of voltage control occurs and the somatic and axonal compartments are effectively decoupled. This phenomenon underlies the fact that voltage-clamp recordings at the soma capture large spikes of inward current when a threshold voltage is exceeded [23].

**Figure 2:**
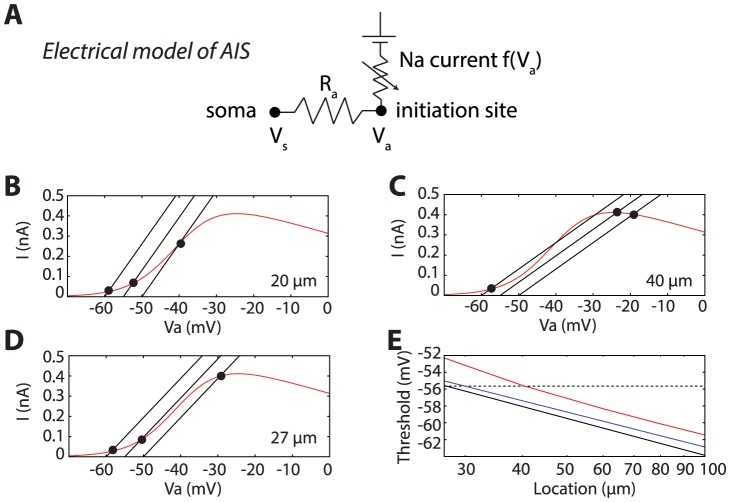
Spike initiation sharpness due to a bifurcation. A, Simplified electrical model of the axon initial segment (used for theoretical analysis but not for simulations). The initiation site is coupled to the soma through an axial resistance Ra. Sodium current f(V_a_) equals axial current (V_a_−V_s_)/R_a_. B, Sodium current (red) and lateral current (black) as a function of voltage at the initiation site when Na channels are placed at 20 µm away from the soma. The three lines correspond to somatic voltages of −60 mV, −55 mV and −50 mV. C, Same as B, with Na channels at 40 µm from the soma. D, Same as A at the critical point, with channels at 27 µm away from the soma. E, Predicted somatic spike threshold, defined as the bifurcation point, as a function of location of the initiation site (logarithmic scale) for the full formula (black) and its approximation (blue). The red curve shows the somatic voltage at which half of the sodium channels are activated in the numerical simulation of the ball-and-stick model. The dashed line is the predicted spike threshold at the critical point.

Mathematically, this loss of voltage control corresponds to a bifurcation, that is, a sudden change in the equilibrium points of the system when a parameter (here somatic voltage) is changed by a small amount. The Na current is a function f(V_a_) of the voltage V_a_ at the initiation site. If the soma acts as a current sink, then the lateral current must be equal to the Na current. This corresponds to a simplified electrical model that approximates the spatially extended model, in which the initiation site and the soma are connected by a resistor, and the Na current is inserted at the initiation site (Fig. 2A). In this section, I analyze the properties of this simplified model, and then I derive theoretical predictions that match numerical simulations of the ball-and-stick model. The lateral current is given by Ohm's law: I = (V_a_−V_s_)/R_a_, where V_s_ is the somatic voltage and R_a_ is the axial resistance between the soma and the initiation site, which is proportional to the distance. Thus at any time the axonal voltage V_a_ is determined by the somatic voltage V_s_ through a non-linear equation, which expresses the equality of the lateral and Na currents: (V_a_−V_s_)/R_a_ = f(V_a_). This equation, which I shall call the current equation, is almost equivalent to the cooperativity model of Na channels [4], [18], and therefore has the same properties. Fig. 2B shows the Na current (red) as a function of V_a_ for an initiation site at 20 µm from the soma, corresponding to the green curves in Fig. 1C–D. The black curves show the lateral current as a function of V_a_ for a somatic voltage V_s_ of −60, −55 and −50 mV. The value of V_a_ is determined by the intersection of the red and black curves: −59, −52 and −40 mV. Thus V_a_ is amplified compared to the somatic voltage but still varies continuously with it. When the initiation site is at 40 µm from the soma, corresponding to the red curves in Fig. 1C–D, a qualitatively different situation occurs, as shown in Fig. 2C. Compared to the previous case, the only difference is that the axial resistance R_a_ is twice larger. But now as the somatic voltage is increased, the intersection point suddenly jumps from about −55 mV to −25 mV. This occurs because the number of solutions to the current equation changes from 3 to 1 when V_s_ is increased, that is, a bifurcation occurs with respect to variable V_s_. It corresponds to the loss of voltage control seen in Fig. 1F.

Graphically, this bifurcation occurs when the line representing the lateral current (black) is tangent to the curve representing the Na current (red) at the intersection point. This can only happen if the slope of the line (1/R_a_) is smaller than the maximum slope of the Na curve. Thus there is a critical value of R_a_ above which spike initiation becomes sharp. At this point, represented in Fig. 2D, the black line is tangent to the red curve at the inflexion point. This critical value can be calculated as a function of parameters (see Text S1, section 1). With the Na channel properties used in this model, the condition for sharpness is approximately: R_a_.g_Na_>0.27, where R_a_ is the axial resistance to the initiation site and g_Na_ is the total maximal conductance of Na channels. The axial resistance is determined by the geometry of the AIS and by the intrinsic resistivity R_i_ (150 Ω.cm in this model). The condition can then be written R_i_.g_Na_.x/d^2^>0.21, where d is the axon diameter and x is the distance of the initiation site away from the soma. For the present model, the critical point occurs when the Na channels are placed at distance x = 27 µm away from the soma (see Fig. 2D).

The spike threshold can be defined as the voltage at the bifurcation point, which is when the line representing the lateral current (black) is tangent to the curve representing the Na current (red) at the intersection point. Mathematically, this is obtained by differentiating the current equation with respect to V_a_: 1/R_a_ = f′(V_a_). A simple calculation shows that the threshold is higher at the initiation site than at the soma by an amount k_a_ (see Text S1, section 2.1), the Boltzmann slope factor of the Na activation curve, which is about 6 mV according to patch-clamp measurements. This is in very close quantitative agreement with dual whole-cell recordings in the soma and AIS of cortical cells [19]. The somatic spike threshold can also be calculated. A full equation is given in the Text S1 (section 2.2), which can be approximated as follows:
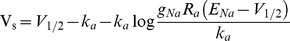
This is similar to the equation derived for an isopotential neuron [21], but a striking difference is that the threshold does not depend on the leak conductance. In effect, the equation is almost identical, with the leak resistance (1/g_L_) replaced by the axial resistance R_a_. This equation implies that the spike threshold decreases logarithmically with the distance of the initiation site. Fig. 2E shows the spike threshold as a function of the distance of the initiation site in logarithmic scale, as given by the full equation (black) and by the above approximate equation (blue). This is compared to the somatic voltage at which half of the sodium channels are activated in the numerical simulation of the ball-and-stick model (red): as predicted, it decreases logarithmically with distance and is only about 2 mV above the predicted values. At the critical distance (27 µm here), the spike threshold is a constant that depends only on Na channel properties, and is independent of geometry (dashed line). It equals −55.6 mV with the chosen parameters (see calculation in the Text S1).

### The initial shape of spikes

**Figure 3:**
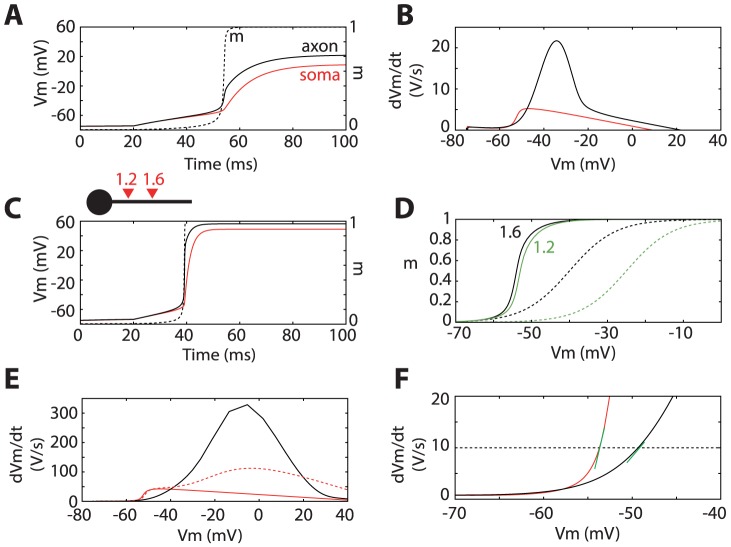
The “kink” at spike onset. A, A pulse of current is injected at time 20(black), with Na channels opening almost all at once (dashed black: proportion of open channels), resulting in a discontinuity in the voltage derivative at the soma (the “kink”). B, Phase plot (voltage derivative vs. voltage) at the initiation site (black) and at the soma (red), showing a (modest) kink at the soma of about 5 mV/ms. C, Same as A, but Nav1.2 channels are placed at 15 µm from the soma, in addition to the Nav1.6 channels at 40 µm. D, Proportion of open Na channels as a function of somatic voltage, for both channel subtypes (Nav1.6 at initiation site, Nav1.2 closer to the soma). The two activation curves are shown in dashed with the same colors. E, Same as B, but with the additional Nav1.2 channels. The dashed red curve shows the somatic phase plot when Nav1.2 channels are also added at the soma. F, Enlargement of panel F, Onset rapidness is measured as the slope of the trajectories (green segments) when dVm/dt reaches 10 mV/ms (dashed line).

Many empirical discussions have focused on the shape of action potentials at onset: a “kink” is indeed observed in cortical neurons, as if the action potential were suddenly rising from nowhere [4], [6]. This kink reflects the lateral current coming from the axon: indeed it can be recorded under somatic voltage clamp [23]. In the present model, Na channels open almost instantaneously after the bifurcation. This produces a discontinuous change in the lateral current, between the initiation site and soma, equal to ΔV/R_a_, where ΔV = V_a_−V_s_ is the voltage difference between the somatic voltage and the voltage at the initiation site, i.e., the discontinuous voltage change seen in Fig. 1F. This quantity can be analytically calculated in the simplified model (see Text S1, section 3), and is about Δ*V*≈33 mV for the case shown in Fig. 1F (consistent with the numerical simulation). The lateral current then jumps to the value ΔV/R_a_ 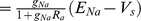. For the present model, with an initiation site at 40 µm, this gives an initial “kink” in the voltage derivative at the soma of dV/dt = ΔV/(CR_a_) = 7.5 mV/ms. Fig. 3A shows the response of the ball-and-stick model to a somatically injected current pulse, both at the initiation site (black) and soma (red). At some point, all Na channels open abruptly (dashed) and as a result, there is indeed a sudden increase in the voltage derivative. The phase plot, representing dV/dt as a function of V, shows that this increase is about 5.2 mV/ms, near the predicted value (Fig. 3B).

Two remarks are in order. First, from the formula, it can be seen that this kink becomes more pronounced when Na conductance increases, which is expected, but it becomes *less* pronounced when the initiation site is moved away from the soma (R_a_ increases). This latter fact is more surprising, because it means that the sharpness of the “kink” at the soma is *inversely* correlated with the sharpness of spike initiation. Second, even though this kink is significant, it remains an order of magnitude smaller than what is typically observed in cortical neurons. Thus it appears in this case that spike initiation can be sharp (abrupt all-or-none opening of Na channels when somatic voltage exceeds a threshold value), without producing a very strong “kink” at the soma. This is because the initial shape of spikes at the soma is not only determined by the sharpness of spike initiation, but also by properties of the piece of axon between initiation site and soma.

Increasing the Na conductance would lower the threshold (by about 4 mV for every doubling), and in any case it cannot push the lateral current above 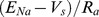, which is the current obtained with a fully developed spike at the initiation site. Thus, to obtain a larger “kink”, there must be a fully developed spike closer to the soma. As we have seen (Fig. 1E), the voltage across the axon increases linearly between the soma and the initiation site when all Na channels are clustered at that point. Therefore no spike can develop closer to the soma unless additional Na channels are also present closer to the soma.

Therefore, to explore the conditions for a significant “kink” at the soma, we now consider Na channels between the soma and initiation site, again clustered at a single location (distributed channels are considered later). Immunostaining in the AIS of cortical neurons shows that low-threshold Nav1.6 channels accumulate at the distal end of the AIS, while high-threshold Nav1.2 channels accumulate at the proximal end [9]. The half-activation voltage of Nav1.2 can be higher than that of Nav1.6 by up to about 15 mV. This implies that the maximum conductance for Nav1.2 can be set an order of magnitude larger than for Nav1.6 without affecting spike initiation or resting potential. When a spike is initiated at the distal end of the AIS, the voltage suddenly rises at the location of Nav1.2 channels. If this shift is sufficient, these channels suddenly open and produce an additional current to the soma. Thus, for this phenomenon to occur, Nav1.2 channels must be placed at an intermediate position between the soma and the initiation site: if they are too close to the soma, the voltage does not increase sufficiently at spike initiation to open the channels; if they are too far from the soma, the lateral current is small. In Fig. 3C, Nav1.2 channels are placed at 15 µm and Nav1.6 channels at 40 µm. Spike initiation is still sharp (Na channels open abruptly) and the spike threshold is similar, but the kink at the soma is much more pronounced. Fig. 3D shows that Nav1.6 channels at the initiation site open abruptly when the soma is depolarized (black), shortly followed by Nav1.2 channels (green). This panel highlights two points: 1) both types of channels open much more abruptly than the activation curves alone would suggest (dashed), 2) Nav1.2 channels open at about the same somatic voltage as Nav1.6 channels, even though there is a 15 mV shift in the activation curves. This latter observation derives from the fact that when Nav1.6 channels open, the voltage at the site where Nav1.2 channels are placed suddenly increases above their bifurcation point. As a result, the voltage derivative at the soma now reaches 42 mV/ms (Fig. 3E), about 8 times higher than without the new channels. Note that there are no Na channels at the soma in this model, which would produce the biphasic trajectory typical of cortical cells (dashed red curve). Additional Na channels at the soma have no impact on initial spike shape at the soma (first “bump” in the plot), because the “kink” reflects the current coming from the axon.

To quantify the initial sharpness of spikes, previous studies have used a measure named “onset rapidness”, defined as the slope of the trajectory in the phase plot when a fixed value α of dV/dt is reached, typically of the order of α = 10 mV/ms [4], [8]. Perhaps surprisingly, despite the fact that Na channels open abruptly at the AIS, this sharpness does not appear in the phase plot, where onset rapidness is low, about 2 ms^−1^ (Fig. 3F, black).This observation may be confusing: on one hand, spike initiation is sharp, but on the other hand the initial shape of spikes is not sharp at the initiation site. Some explanation is necessary. That spike initiation is sharp means that Na channels in the initiation site open abruptly as a function of the *somatic* membrane potential V_s_, and as a function of time. But as a function of the *axonal* membrane potential V_a_ at the initiation site, the opening of Na channels follows the Na activation function 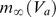, which does not depend on spatial properties. Therefore the derivative dV_a_/dt essentially reflects the Na activation function, and “onset rapidness” at the initiation site is essentially a measure of this function. This point can be demonstrated analytically: at the initiation site, as in an isopotential neuron, onset rapidness equals α/k_a_, independently of all other properties (Text S1, section 4.1). In the present model, this theoretical prediction is 10 mV.ms^−1^/6 mV≈1.7.ms^−1^, close to the numerical value. In contrast, onset rapidness is about four times larger at the soma (7.7 ms^−1^). Indeed the voltage trajectory at the soma is determined by the lateral current, and in particular should correlate with the total Na conductance at the initiation site (Text S1, section 4.2). This difference between soma and AIS is consistent with patch recordings in the soma and axon of the same cells [5], [8].

As this is a rather subtle point, I will try to rephrase this result, in the context of previous results. At the initiation site, the voltage derivative dV_a_/dt reflects the Na current, and therefore is a smooth function of V_a_, as determined by the Na activation curve (Fig. 3E–F). However, as a function of membrane potential V_s_ at the soma, Na channels open abruptly (Fig. 3D). This is because axonal voltage V_a_ is a discontinuous function of somatic voltage V_s_, due to loss of voltage control (Fig. 1F). The “kink” at the soma is a direct consequence of this discontinuity: since it reflects the lateral current, it is proportional to the spatial derivative of voltage along the axon. In summary, sharpness of spike initiation is due to compartmentalization (loss of voltage control); the “kink” at the soma is due to compartmentalization together with transmission by proximal Na channels. Spike initiation can be sharp with only a small kink seen at the soma, if there are no Na channels close to the soma to transmit the spike (Fig. 3B).

### Spatially distributed channels

**Figure 4:**
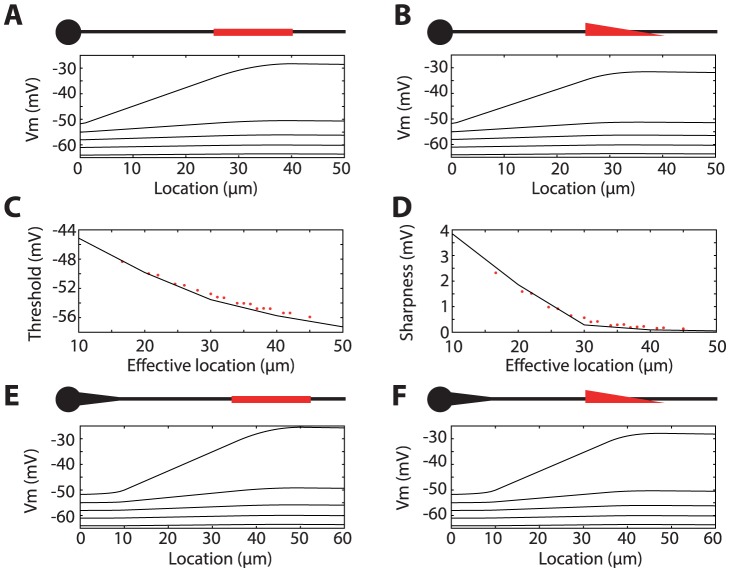
Spatially distributed channels. A, Voltage across the axon as the soma is depolarized by steps of 3(5 values from −64 mV to −52 mV), with Na channels uniformly distributed between 25 and 40 µm away from the soma (compare with Fig. 1E, same total Na conductance). B, Same as A, but with Na channel density linearly decreasing between 25 and 40 µm. C, Spike threshold defined as the somatic voltage at which half of the sodium channels are activated as function of the location of Na channels when they are clustered at a single point (black). Red dots show the spike threshold when Na channels are uniformly distributed on a segment, as a function of the “effective” location, defined as a point inside the segment (x = 0.6 x_start_+0.4 x_end_). Start points are taken among 1, 10, 20, 25, 30 and 35 µm; end points are taken among 40, 50 and 60 µm. D, Same as C but for initiation sharpness. E, Same as A but a tapering piece of axon is inserted at the beginning (the initial segment is moved), with length 10 µm and diameter linearly decreasing from 4 µm to 1 µm. F, Same as B with the initial tapering.

All the previous results were obtained with channels clustered at a single location, but Na channels are rather distributed along the AIS [9]. Analytical formulae are more difficult to obtain in this case, but the same phenomenon occurs. First, when Na channels are continuously distributed on a portion of the AIS, maximum depolarization always occurs at the distal end (Fig. 4A). This is because almost all current flows towards the soma, and therefore the potential must be an increasing function of the distance to the soma. In addition, the spatial voltage profile along the axon is concave, because at any given point, the current flowing towards the soma is always greater than the current coming from the distal end since it also includes the Na current flowing through the membrane at that point. In cable theory, these remarks correspond to the fact that the diffusion current equals the opposite of the Na current at any point (Text S1, section 5). Importantly, these facts hold independently of the particular profile of the Na channel density. For example, if Na channels have linearly decreasing density between 20 µm and 40 µm, spikes are still initiated at the distal end, 40 µm away from the soma (Fig. 4B), which is consistent with recent findings [24]. However, for spike initiation, the situation is not equivalent to the case when all channels are clustered 40 µm away from the soma. Indeed, because the voltage profile is concave, it can be seen that it is in fact close to the one obtained with channels clustered at an intermediate location between the two ends. To show this effect quantitatively, spike threshold and sharpness were calculated with Na channels evenly distributed on a portion of the AIS, with various start and end points, and otherwise the same parameter values as previously (Fig. 4C, D). The total Na maximal conductance was left unchanged, the start point x_1_ varied between 1 µm and 35 µm and the end point x_2_ varied between 40 and 60 µm. Empirically, it appeared that both spike threshold (Fig. 4C) and sharpness (Fig. 4D) corresponded to the values obtained when Na channels are clustered at a single “effective” location x = 0.6 x_1_+0.4 x_2_. This formula was empirically determined and may depend on other parameters.

In the ball-and-stick model, the axon is geometrically modelled as a cylinder. However, at the hillock near the soma, the diameter is larger than in the initial segment [25]. Fig. 4E–F show the effect of inserting a 10 µm tapering piece of axon at the beginning, with diameter linearly decreasing from 4 µm to 1 µm. Since in this model there are no Na channels on this part of the axon, the main effect is to increase the axial resistance R_a_, by an amount that can be analytically calculated (see Text S1, section 1). In this case, it is equivalent to extending the cylindrical axon by 2.5 µm, so the phenomenon is essentially unchanged. However, the voltage increases much more slowly along the axon in this tapering part, which could have consequences if voltage-gated channels are placed in this region.

### Energetic efficiency

It has been proposed that a potential benefit of spike initiation in the distal AIS is to make it more energetically efficient, because the AIS has a smaller capacitance than the soma and therefore requires less transfer of charge to produce a spike, consistently with the fact the current threshold is lower in the axon than in the soma [19]. Indeed energy consumption is essentially proportional to the number of Na ions entering the cell [26]. However, the argument does not apply if a full spike is also seen at the soma, as in cortical cells. In this case, the total transfer of charge carried by Na ions should be about C.ΔV, where C is the total capacitance of the cell where a full spike develops, and ΔV≈100 mV is the spike height (assuming no overlap with outward currents). It is not obvious why the location of the initiation site should make a difference.

**Figure 5:**
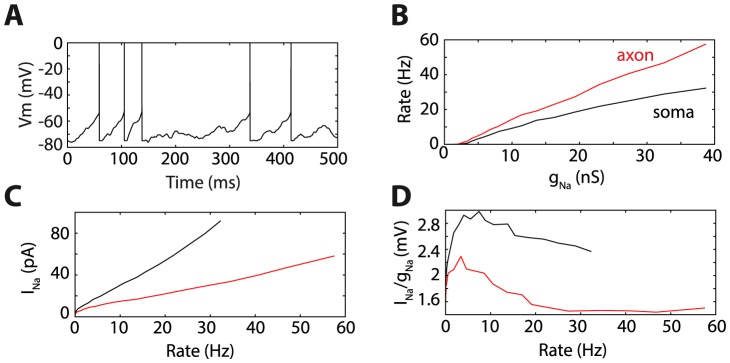
Energy consumption with spike initiation at the soma and in the axon. A, Response of the neuron model with Na channels at 40 µm away from the soma to a fluctuating current injected at the soma. Since there are only Na channels in the model, the membrane potential is reset when half of Na channels are open (spikes are added to the trace for readability). B, Firing rate as a function of maximum Na conductance, when channels are in the axon (red, 40 µm from the soma) and at the soma (black). C, Average Na current as a function of output firing rate, for both cases. D, Same as C, but normalized by the maximum Na conductance (essentially reflecting the average proportion of open channels).

In fact, spike initiation in the distal AIS is indeed more energetically efficient, not because of the smaller axonal capacitance, but because it reduces the flow of Na current below threshold, which is proportional to the rate of ATP consumption [26]. First, the maximal Na conductance required to initiate spikes at a given threshold is inversely proportional to the distance of the initiation site from the soma. Therefore, the Na current at rest is also inversely proportional to that distance. Second, because Na channels open abruptly when initiation is distal, most channels are closed before initiation. To demonstrate these points, the ball-and-stick model is simulated in current-clamp with fluctuating current in two configurations: Na channels at the soma, and at 40 µm away from the soma (Fig. 5). Since only Na channels are included in the model, the membrane potential across the neuron is reset to the resting value when half of the Na channels are open (Fig. 5A). The input current is the same in all simulations, so that subthreshold activity is comparable. Fig. 5B shows the neuron's firing rate as a function of maximal Na conductance: the relationship is approximately linear, but the firing rate increases about twice faster with conductance when Na channels are in the distal AIS, compared to the soma. For the same firing rate, the average Na current is much higher when Na channels are in the soma (Fig. 5C). This difference is indeed not only due to the difference in maximal Na conductance (i.e., number of channels), because this average current is still about twice higher with channels at the soma after it is normalized by the maximal conductance (Fig. 5D): this reflects the fact that when Na channels are at the soma, a substantial proportion of them can open without triggering a spike. Thus the sharpness of spike initiation, and not just the smaller capacitance of the AIS, makes spiking more energetically efficient.

## Discussion

The claim that spike initiation is much sharper in cortical neurons than expected from isopotential Hodgkin-Huxley models is supported by different lines of evidence: 1) the initial shape of spikes recorded at the soma is very sharp, with a distinct “kink” [4], 2) cortical neurons can transmit high frequency signals and follow input changes at the millisecond timescale [11], [12], 3) current-voltage relationships measured at the soma *in vitro* show an effective slope factor of about 1 mV [15], 4) spiking responses of cortical neurons to noisy currents injected at the soma are very well predicted by integrate-and-fire models [16], and less accurately predicted by models with smooth initiation [17].

Two hypotheses have been proposed to explain this phenomenon. One is that Na channels cooperate in the AIS, which would make their collective activation curve much sharper [4]. Cooperative activation has been demonstrated in calcium [27], [28], potassium [29] and HCN channels [30], and in pharmacologically altered Na channels of cardiac myocytes [31]. However, this hypothesis is not supported by direct experimental evidence in Na channels of the AIS, and recordings in axonal blebs found no sign of cooperativity [9]. In addition, in the cooperativity model, the spike threshold appears as a bifurcation in the collective Na activation curve and therefore only depends on Na channel properties, but several studies have found that Kv1 channels, expressed in the AIS, can modulate the spike threshold [32], [33]. The second hypothesis is that the “kink” at spike onset reflects the lateral current coming from the axon, which becomes sharper through the active backpropagation from the initiation site to the soma, while initiation is smooth at the initiation site [5], [8]. Indeed the kink reflects the lateral current coming from the axon [23], and onset rapidness measured at the initiation site is compatible with standard isopotential Hodgkin-Huxley models. However, this hypothesis fails to explain why spike initiation, and not only the initial shape of spikes observed at the soma, is sharp. In addition, the initiation site is very close to the soma, compared to the electrotonic length of the axon (on the order of 5%).

I have proposed a parsimonious explanation of the sharpness of both spike initiation and somatic spike shape, which is compatible with standard biophysics and empirical measurements. The explanation is based on compartmentalization. Because the soma is large compared to the axon diameter, it acts as a current sink for the axonal initiation site. If follows that the Na current equals the resistive axial current at all times, which results in an instability when a voltage threshold is crossed (a bifurcation in dynamical systems theory). This instability manifests itself as a loss of voltage control between the soma and the initiation site. As a result, Na channels open abruptly as a function of somatic voltage. The mathematics of this phenomenon are very close to the cooperativity model, although channels are independent. Spike initiation sharpness does not require active backpropagation, but the “kink” at spike onset at the soma requires proximal Na channels to transmit the spike (Nav1.2).

This simple hypothesis accounts for a number of empirical observations: 1) spike initiation is sharp, even though the “onset rapidness” measure is low at initiation site [5], that is, the voltage derivative at initiation site is a smooth function of membrane potential at that same point; 2) there is an approximately 6 mV difference in spike threshold between the soma and the initiation site [19]; 3) spike initiation occurs distally, but there are higher threshold channels (Nav1.2) closer to the soma in cortical neurons [9]. In addition, according to this hypothesis, spike initiation can be sharp without producing a distinct “kink” at the soma. In neurons of the medial superior olive of gerbils, which are involved in the detection of microsecond timing differences in sound waves, action potentials become smaller and smoother one week after hearing onset, while all other temporal properties (e.g. EPSP halfwidth) are refined [34]. The same pattern is seen in the superior paraolivary nucleus of the mouse [35]. The compartmentalization hypothesis suggests that in those neurons spike initiation remains sharp with development, while only the initial shape of spikes at the soma becomes smooth, through down-regulation of proximal Na channels and possibly up-regulation of K channels. The hypothesis also predicts that the spike threshold should be dynamically modulated through the inactivation of Na channels, but it is also consistent with an effect of Kv1 on the spike threshold [32], [33]. Indeed, channels open at the initiation site (but not at the soma) introduce an additional current that shifts the somatic spike threshold by an amount approximately proportional to the channel conductance (see Text S1, section 6).

What is the functional benefit of spike initiation in the axon? Since in cortical neurons a full spike is also seen at the soma, it cannot be the sole fact that the capacitance of the AIS is lower than that of the soma. In fact, spike initiation in the AIS is more energetically efficient because fewer Na channels are open below threshold than if spikes were initiated at the soma. This is in line with other known mechanisms that enhance the efficiency of action potential propagation [36]. There may be additional benefits related to compartmentalization, for example the fact that inhibitory neurons can directly control the output of neurons by targeting the AIS.

Incidentally, the fact that spike initiation is very sharp and that Na channels open abruptly above a critical voltage has important implications for neural modeling. Indeed, it is commonly assumed that biophysical models of the Hodgkin-Huxley family are more realistic than simpler phenomenological models such as the integrate-and-fire model, and that the latter are only used for their computational and theoretical simplicity. However, these results show that the simplified model shown in Fig. 2A is a better approximation of spike initiation in the spatially extended model than a single-compartment Hodgkin-Huxley model (shown in Fig. 1A–B). But that model is essentially an integrate-and-fire model when Na channels are far enough in the axon (Fig. 1C–D). Therefore, as far as single-compartment models are concerned (not biophysically realistic multicompartmental models), the integrate-and-fire model is in fact more accurate than models with smooth spike initiation, including single-compartment Hodkgin-Huxley models.

## Methods

### Numerical simulations and models

All neuron models were simulated with the Brian simulator [37], with temporal resolution 25 µs and spatial resolution 1 µm. The ball-and-stick model consists of a sphere of diameter 50 µm, representing the soma, and a cylinder of diameter 1 µm and length 300 µm, representing the axon. Note that the electrical model shown in Fig. 2A is only used for analytical calculations (see Text S1), but all simulations use the ball-and-stick morphology. Specific membrane resistance is R_m_ = 30,000 Ω.cm^2^, specific membrane capacitance is C_m_ = 0.75 µF.cm^−2^, giving a membrane time constant of 22.5 ms. Intracellular resistivity is R_i_ = 150 Ω.cm. The leak reversal potential is E_L_ = −75 mV. Apart from leak channels, only Na channels were included, so as to demonstrate the phenomenon without confounding factors (see Text S1 for the impact of Kv1 channels). The channels did not inactivate, so the Na channels in the model represent the available channels. In most simulations, Na channels are clustered at a single location in the axon. The Na current is defined by the following equations:
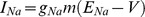


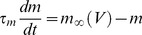


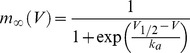
where E_Na_ = 60 mV, k_a_ = 6 mV, V_1/2_ = −40 mV, τ_m_ = 100 µs [38]. Note that the current equation (I_Na_) uses m^1^ rather than m^3^, which is used in the original Hodgkin-Huxley model. Indeed recent evidence shows that this is more accurate for Na channels of central neurons in mammalians [39]. It also makes it easier to relate the parameters of the activation curve with patch-clamp measurements.

Unless specified, the maximum total Na conductance g_Na_ is twice the somatic leak conductance. Note that, since the model did not include inactivation, this represents the conductance of available channels (i.e., maximal conductance would be larger). The model is simulated in somatic voltage-clamp mode in Figs. 1, 2 and 4, and in somatic current-clamp in Figs. 3 and 5.

In Fig. 3, Nav1.6 channels are placed at 40 µm away from the soma, and additional Nav1.2 channels are placed at 15 µm from the soma. The activation curve of Nav1.2 is depolarized by 15 mV, compared to Nav1.6 channels (i.e., V_1/2_ = −25 mV), and the maximal conductance is 20 times larger than for Nav1.6 channels (again this represents the ratio for available channels; considering that Nav1.6 channels are partially inactivated, the ratio would be lower). In Fig. 3E, dashed curve, somatic Nav1.2 channels are also added, with the same total conductance as in the axon. In Fig. 4, Nav1.6 channels are spatially distributed (uniformly in A or linearly in B) with the same total conductance as before.

In Fig. 5, a fluctuating current is injected into the soma, according to the following equation:
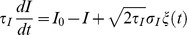
where τ_I_ = 5 ms, I_0_ = 18.3 pA, σ_I_ = 31.4 pA and 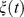 is Brownian noise. The current is identical in all simulations, only the total Na conductance varies. Since only Na channels are included in the model, spikes are triggered when half of the Na channels are open, then the membrane potential is reset to the leak reversal potential across the neuron, and clamped at that value for 5 ms.

### Theoretical model

Theoretical calculations are based on a simplified model of the axon initial segment (AIS). The analysis is described in more detail in the supplementary methods (Text S1). The equivalent electrical circuit is shown in Fig. 2A. Na channels are placed in the initiation site only. The initiation site receives a Na current I_Na_ = f(V_a_) as specified in the previous section, where V_a_ is the voltage at the initiation site (*a* for axonal), and is electrically coupled to the soma by the axial resistance R_a_. In this model, the lateral current equals the Na current, which means:
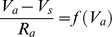
Thus the axonal voltage V_a_ is determined as an implicit function of the somatic voltage V_s_ through a fixed point equation. This equation exhibits a bifurcation when R_a_ is above a critical value determined by model parameters, in particular by the geometry (Text S1, section 1). This means that, when there is a bifurcation, the solution V_a_ to this equation changes discontinuously (discrete increase) when the somatic voltage V_s_ exceeds a certain value (the bifurcation point). We define the spike threshold as this value.

The spike threshold can be calculated as the bifurcation point of the above equation, as a function of model parameters (Text S1, section 2). The calculation shows that, at threshold, 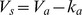 (i.e., a difference of about 6 mV between the two sites), and V_s_ is given by the following approximated formula:
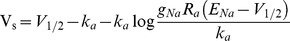



## Supporting Information

Text S1 **Supplementary methods.** These supplementary methods describe a theoretical analysis of spike initiation in a simplified model of the axon initial segment (AIS).
(PDF)
