# Supplementary material for: Sharpness of Spike Initiation in Neurons Explained by Compartmentalization
Source: PLoS Comput Biol. 2013 Dec 5;9(12):e1003338. doi: 10.1371/journal.pcbi.1003338 (PMC3854010; doi:10.1371/journal.pcbi.1003338)
Supplement: Text S1 — Supplementary methods. These supplementary methods describe a theoretical analysis of spike initiation in a simplified model of the axon initial segment (AIS). (PDF) [file pcbi.1003338.s001.pdf]

Supplementary Text S1

Sharpness of spike initiation in neurons explained by compartmentalization

Romain Brette (romain.brette@ens.fr)

These supplementary methods describe a theoretical analysis of spike initiation in a simplified model of the axon initial segment (AIS).

1. Bifurcation in the electrical model of the axon initial segment (AIS)

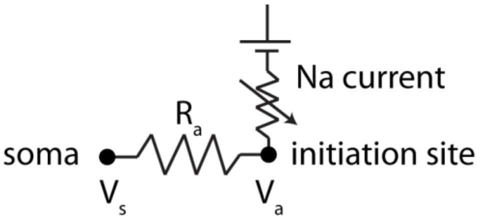

Na channels are placed in the initiation site only. The initiation site receives a Na current $f(V_a)$, where V_a is the voltage at the initiation site (*a* for axonal), and is electrically coupled to the soma by the axial resistance R_a. This resistance is determined by cable theory through the following formula:

$$R_a = \frac{4R_i}{\pi d^2}\text{x}$$

where R_i is intracellular resistivity (R_i = 150 Ω.cm here), d is the diameter of the axon (d = 1 µm here) and x is the distance to the soma (typically about 40 µm). This simplification is justified as follows:

- The initiation site is electrotonically close to the soma, about 40 µm compared to a space constant of magnitude 1 mm (λ = 700 µm here), so a single compartment is sufficient.
- For the same reason, the current to the distal end of the AIS is negligible compared to the lateral current to the soma (see Fig. 1E).
- The Na current activates very quickly (characteristic time constant is a fraction of ms) and therefore the proportion of open channels is considered equal to its equilibrium value.
- Capacitive currents are small, given the small area of the AIS.

Tapering in the proximal part of the axon (hillock) can be taken into account by modifying the axial resistance R_a. If axonal diameter decreases linearly over an initial length y from d(0) to d(y), then the axial resistance of that part of axon is (resistance of a truncated cone):

$$R_a^0 = \frac{4R_i}{\pi d(0)d(y)}\text{y}$$

It can then be added to the axial resistance of the rest of the axon (formula above).

In this model, the lateral current equals the Na current, which means:

$$\frac{V_a - V_s}{R_a} = f(V_a)$$

Thus the axonal voltage V_a is determined as an implicit function of the somatic voltage V_s through a fixed point equation. This function is plotted in Fig. 1F. At a critical voltage, V_a suddenly changes. This corresponds to a bifurcation, when the solution of the above equation becomes unstable. The value of this critical point is obtaining by differentiating the equation:

$$1 = R_a f'(V_a)$$

The value of V_a that satisfies this equation is the axonal threshold (see below). For a bifurcation to exist, the above equation must have a solution, which will depend on R_a. To get a quantitative estimate, we now consider that the Na current is given by the following equation:

$$f(V_a) = g_{Na}(E_{Na} - V_a)h(\frac{V_a - V_{1/2}}{k_a})$$

where h is the unit Boltzmann function and $V_{1/2}$ is the half-activation voltage. The Boltzmann function satisfies: h'=h(1-h).

Thus the derivative of the current is:

$$f'(V_a) = -g_{Na} h\left(\frac{V_a - V_{1/2}}{k_a}\right) + g_{Na}\frac{E_{Na} - V_a}{k_a} h\left(\frac{V_a - V_{1/2}}{k_a}\right)(1 - h\left(\frac{V_a - V_{1/2}}{k_a}\right))$$

There is a bifurcation if the maximum of f'(V) is greater than $1/R_a$. However, there is no simple analytical formula for this maximum. As an approximation, we consider the value at $V=V_{1/2}$:

$$f'(V_{1/2}) = -\frac{g_{Na}}{2} + \frac{g_{Na}}{4}\frac{E_{Na} - V_{1/2}}{k_a}$$

Then we have:

$$R_a f'(V_{1/2}) = R_a g_{Na}\left(-\frac{1}{2} + \frac{E_{Na} - V_{1/2}}{4k_a}\right)$$

If this is greater than 1, then there is bifurcation. This gives a condition on $R_a.g_{Na}$. Using: E_{Na} = 60 mV, $V_{1/2}$ = -40 mV, k_a = 6 mV, we get the following condition:

$$R_a g_{Na} > 0.27$$

This can be related to the geometry of the AIS. If it is assumed that Na channel density is uniform over length Δ, then:

$$g_{Na} = g^* \Delta \pi d$$

where g* is the Na channel density. With the same numbers as above, the bifurcation condition reads:

$$g^* R_i \frac{\mathrm{x}\Delta}{\mathrm{d}} > 0.07$$

In other words, there is a critical distance from the soma for the initiation site, above which spike initiation is sharp.

2. Spike threshold

We now estimate the value of the voltage at the bifurcation point, which we define as the spike threshold. We may define the threshold at the soma (for V_s) or at the initiation site (for V_a).

2.1. Axonal and somatic threshold

To this end, we make the assumption that the bifurcation occurs when V_a is well below $V_{1/2}$, so that the activation curve of the Na channels (a Boltzmann function) can be approximated by an exponential function (and of course, we make the assumption that there is a bifurcation). We obtain the following equation (axial current equals Na current):

$$\frac{V_a - V_s}{R_a} = g_{Na}(E_{Na} - V_a)\exp(\frac{V_a - V_{1/2}}{k_a})$$

This can be rewritten as follows:

$$V_a - V_s + g_{Na}R_a(V_a - E_{Na})\exp\left(\frac{V_a - V_{1/2}}{k_a}\right) = 0 \qquad (1)$$

At the bifurcation point, we have (differentiate with respect to V_a):

$$1 + g_{Na}R_a\exp\left(\frac{V_a - V_{1/2}}{k_a}\right)(1 + \frac{V_a - E_{Na}}{k_a}) = 0$$

Given that $|V_a\text{-}E_{Na}| >> k_a$, this simplifies to:

$$g_{Na}R_a\exp\left(\frac{V_a - V_{\frac{1}{2}}}{k_a}\right)\left(\frac{V_a - E_{Na}}{k_a}\right) = -1 \qquad (2)$$

We combine it with equation (1), and obtain:

$$V_s = V_a - k_a$$

Therefore, there is a difference $k_a \approx 6$ mV between the somatic and axonal threshold.

2.2. The threshold equation

An approximated equation for the axonal threshold is obtained by replacing $E_{Na}\text{-}V_a$ by $E_{Na}\text{-}V_{1/2}$ in equation (2):

$$V_a = V_{1/2} - k_a\log\frac{g_{Na}R_a(E_{Na} - V_{1/2})}{k_a}$$

Thus the spike threshold at the soma is:

$$\mathrm{V_s} = V_{1/2} - k_a - k_a \log\frac{g_{Na}R_a(E_{Na} - V_{1/2})}{k_a}$$

It is possible to obtain a more accurate threshold equation, from equation (2), using a special function. The Lambert function is defined as the solution x=W(y) to y=xe^x. We rewrite equation (2) as follows:

$$\frac{V_a - E_{Na}}{k_a}\exp\left(\frac{V_a - E_{Na}}{k_a} + \frac{E_{Na} - V_{1/2}}{k_a}\right) = -\frac{1}{g_{Na}R_a}$$

Writing x=(V_a-E_{Na})/k_a, we get:

$$xe^x = -\frac{1}{g_{Na}R_a}\exp\left(\frac{V_{1/2} - E_{Na}}{k_a}\right)$$

There are in fact two solutions for negative numbers. Here clearly x<-1, and therefore we are interested in the lower branch W_{-1} of the Lambert function. Therefore, we obtain:

$$V_a = E_{Na} + k_a W_{-1}\left(-\frac{1}{g_{Na}R_a}\exp\left(\frac{V_{1/2} - E_{Na}}{k_a}\right)\right)$$

and the spike threshold at the soma is:

$$V_s = E_{Na} - k_a + k_a W_{-1}\left(-\frac{1}{g_{Na}R_a}\exp\left(\frac{V_{1/2} - E_{Na}}{k_a}\right)\right)$$

At the critical point (section 1), we have:

$$R_a g_{Na}\left(-\frac{1}{2} + \frac{E_{Na} - k_a - V_{1/2}}{4k_a}\right) = 1$$

And therefore:

$$V_s = E_{Na} - k_a + k_a W_{-1}\left(-\left(-\frac{1}{2} + \frac{E_{Na} - k_a - V_{1/2}}{4k_a}\right)\exp\left(\frac{V_{1/2} - E_{Na}}{k_a}\right)\right)$$

This is the maximum spike threshold when initiation is sharp, and it is a constant that is independent of geometry.

3. The kink

At the soma, a lateral current is received at spike initiation, after the bifurcation occurs. When all Na channels are open, the fixed point equation reads:

$$V_a = V_s + g_{Na}R_a(E_{Na} - V_a)$$

This defines V_a as a function of V_s:

$$V_a = \frac{V_s + g_{Na}R_aE_{Na}}{1 + g_{Na}R_a} = \frac{V_s - E_{Na}}{1 + g_{Na}R_a} + E_{Na}$$

The voltage difference between soma and initiation site is then:

$$\Delta V = V_a - V_s = \frac{g_{Na} R_a}{1 + g_{Na} R_a}(E_{Na} - V_s)$$

The lateral current is then:

$$I_l = \frac{\Delta V}{R_a} \approx \frac{g_{Na}}{1 + g_{Na} R_a}(E_{Na} - V_s)$$

Note that the largest possible current (with large g_{Na}) is $(EN_a\text{-}V_s)/R_a$, and therefore is inversely proportional to the distance between the initiation site (or a proximal site in the axon with Na channels) and the soma.

4. Onset rapidness

4.1. Onset rapidness at the axonal initiation site

Onset rapidness has been characterized as the slope of the spike trajectory in phase plot (dV/dt vs. V) when dV/dt has a specific value α (typically 10 mV/ms). This corresponds to $(d^2V/dt^2)/(dV/dt)$ at that point, which equals d/dt(log dV/dt).

We consider the membrane equation at the axonal initiation site, neglecting resistive currents:

$$C\frac{dV_a}{dt} = g_{Na}\exp\left(\frac{V_a - V_{\frac{1}{2}}}{k_a}\right)(E_{Na} - V_a)$$

where C is the capacitance of the axonal compartment.

Then:

$$\frac{d}{dt}\left(\log\frac{dV_a}{dt}\right) = \left(\frac{1}{k_a} - \frac{1}{E_{Na} - V_a}\right).\frac{dV_a}{dt}$$

At spike initiation, $E_{Na} - V_a >> k_a$, and therefore onset rapidness is:

$$\frac{d}{dt}\left(\log\frac{dV_a}{dt}\right) \approx \frac{1}{k_a}.\frac{dV_a}{dt}$$

Therefore, with k_a = 6 mV and dV_a/dt = 10 mV/ms, we get onset rapidness of about 1.7 ms^{-1}. This calculation would be identical with a single-compartment (isopotential) neuron.

4.2. Onset rapidness at the soma

Since the spike is initiated in the axon, the initial "kink" in the voltage at the soma essentially reflects the lateral current, that is:

$$C\frac{dV_s}{dt} = \frac{V_a - V_s}{R_a}$$

where V_s is the somatic voltage and V_a is the axonal voltage. Therefore, a criterion α = dV_s/dt corresponds to a specific value of the voltage mismatch between soma and AIS:

$$V_a - V_s = R_a C \alpha$$

With previous parameters, this corresponds to 45 mV for Na channels at 40 µm, and to 17 mV with channels at 15 µm.

We differentiate the membrane equation:

$$R_a C \frac{d^2 V_s}{dt^2} = \frac{dV_a}{dt} - \alpha$$

That is:

$$R_a C \frac{d^2 V_s}{dt^2} = \frac{g_{Na}}{C_a} \exp\left(\frac{V_a - V_{\frac{1}{2}}}{k_a}\right)(E_{Na} - V_a) - \alpha$$

where C_a is the capacitance of the axonal compartment.

Dividing by Ra.C.dV/dt (=Ra.C.α):

$$\frac{d^2 V_s}{dt^2} / \frac{dV_s}{dt} = \frac{g_{Na}}{\alpha R_a C C_a} \exp\left(\frac{V_a - V_{\frac{1}{2}}}{k_a}\right)(E_{Na} - V_a) - \frac{1}{R_a C}$$

with $V_a = V_s + R_a C \alpha$ (where V_s is close to threshold). It appears that onset rapidness correlates with the total Na conductance, unlike the isopotential case.

5. Distributed channels

We now consider that Na channels are distributed with density u(x) along the AIS. Neglecting capacitive and leak currents on the AIS, the cable equation reads:

$$\lambda^2 \frac{d^2 V}{dx^2} = -u(x) f(V)$$

with two boundary conditions: V(0)=V_s and dV/dt(L)=0, where L is the distal end of the AIS and f(V) is the proportion of open channels times (E_{Na}-V). The solution of this second-order equation corresponds to the voltage profile across the axon shown in Fig. 4A-B. This equation implies that the profile is concave (voltage derivative decreases as x increases), and since the minimum derivative is reached at the end x = L and equals 0, it must be positive on the entire AIS. Therefore the voltage increases along the axon, even if the channel density decreases or is non-monotonous.

In principle, it is possible to define a bifurcation problem in this setting, but it is a non-standard one as the bifurcation parameter is a boundary condition (the somatic voltage).

6. Kv1 channels

6.1. Bifurcation condition

Low-threshold Kv1 channels are also expressed in the AIS. Here we consider that they are expressed at the same location as Nav1.6 channels, clustered at a single point in the AIS. The bifurcation condition is unchanged:

$$1 = R_a f'(V_a)$$

but the current $f(V_a)$ now includes the Kv1 current:

$$f(V_a) = g_{Na}(E_{Na} - V_a)h\left(\frac{V_a - V_{\frac{1}{2}}}{k_a}\right) + g_K(E_K - V_a)$$

where g_K is the Kv1 conductance and E_K is the reversal potential. Thus the condition for sharp spike initiation becomes:

$$R_a f'(V_{1/2}) = R_a g_{Na}\left(-\frac{1}{2} + \frac{E_{Na} - V_{1/2}}{4k_a}\right) - R_a g_K > 1$$

Therefore, the phenomenon is essentially unchanged, but there are quantitative differences.

6.2. Axonal and somatic threshold

Regarding the spike threshold, equation (1) (section 2.1) becomes:

$$V_a - V_s + g_{Na}R_a(V_a - E_{Na})\exp\left(\frac{V_a - V_{\frac{1}{2}}}{k_a}\right) + g_K R_a(V_a - E_K) = 0 \qquad (1^*)$$

At the bifurcation point, we have (differentiate with respect to V):

$$1 + g_K R_a + g_{Na}R_a \exp\left(\frac{V_a - V_{1/2}}{k_a}\right)(1 + \frac{V_a - E_{Na}}{k_a}) = 0$$

Given that $|V_a\text{-}E_{Na}| >> k_a$, this simplifies to:

$$g_{Na}R_a \exp\left(\frac{V_a - V_{\frac{1}{2}}}{k_a}\right)\left(\frac{V_a - E_{Na}}{k_a}\right) = -1 - g_K R_a \qquad (2^*)$$

As previously, we replace $E_{Na}\text{-}V_a$ by $E_{Na}\text{-}V_{1/2}$ in equation (2*):

$$\mathrm{V_a} = V_{1/2} - k_a \log\frac{g_{Na}R_a(E_{Na} - V_{1/2})}{k_a(1 + g_K R_a)}$$

This is the spike threshold at the initiation site. As expected, the threshold increases when the Kv1 conductance increases. This equation is in fact essentially identical to the one derived in isopotential neurons, with the leak conductance replaced by the inverse of the axial resistance (Platkiewicz & Brette, PLoS Comp. Biol. 2010). In particular, the spike threshold increases logarithmically with the conductance at the initiation site.

We combine equations (1*) and (2*) to obtain the somatic threshold:

$$V_s = V_a - k_a + g_K R_a (V_a - E_K - k_a)$$

where V_a is given by the above equation. This predicts that the difference between the somatic and axonal threshold should increase when Kv1 channels open. Thus, the somatic spike threshold increases approximately *linearly* (and not logarithmically) with Kv1 conductance. Therefore, a relatively small amount of Kv1 current is sufficient to depolarize the somatic spike threshold.

6.3. Energetic efficiency

Since Kv1 channels oppose the currents produced by Na channels, it may seem that they are an energetically expensive way of modulating the spike threshold. However, the additional energy cost is limited.

The Na current is:

$$I_{Na} = g_{Na} \exp\left(\frac{V_a - V_{\frac{1}{2}}}{k_a}\right)\left(\frac{E_{Na} - V_a}{k_a}\right)$$

and from equation (2*) we find that its value at spike initiation is:

$$I_{Na} = \frac{k_a}{R_a}(1 + g_K R_a)$$

This current increases in an affine way with the Kv1 conductance, and therefore (approximately) with somatic spike threshold. To give an order of magnitude, if the spike threshold with all Kv1 channels closed is -60 mV and E_K = -90 mV, then the Na current at spike initiation is doubled for a spike threshold shift of 36 mV ($g_K.R_a = 1$), which is about 3 times larger than the maximal span typically observed in cortical neurons in vivo.
